# General Internet service assessment by latency including partial measurements

**DOI:** 10.7717/peerj-cs.1072

**Published:** 2022-08-19

**Authors:** Dan Komosny

**Affiliations:** Department of Telecommunications, FEEC, Brno University of Technology, Czech Republic

**Keywords:** Internet, Service, Latency, Measurement, IP address, CDN, ISP, E-commerce

## Abstract

Latency is one of the key parameters of Internet services. However, it is difficult to correctly assess a service by its latency. Many latency measurements are blocked en route by routers and firewalls. For this reason, the service latency is not fully known. This work proposes a method to assess Internet services including the blocked latency measurements. Survival theory is applied to process latency values. The results show that the omission of blocked latencies from statistical processing severely underestimates the service latency. Two Internet service providers were compared as an example. Their latency difference was 9 ms using the traditional approach. The survival latency resulted in a difference of 17 ms. The method of survival latency can be used to increase revenues in e-commerce and to improve the experience of online gaming.

## Introduction

There are many types of Internet services, including content delivery network (CDN), software as a service (SaaS), platform as a service (PaaS), Infrastructure as a service (IaaS), and ISP (Internet service provider) connection service. The quality of Internet services is assessed by various measures. These include latency, which is a crucial service parameter. Service latency is mainly given by the geographical distribution of the service cache servers, backbone connection, and peering to other networks. It is stated that latency is the key goal for future Internet development (Singla et al., 2014; Douch et al., 2022).

This work deals with service assessment by latency, which is difficult to correctly implement. Service latency is assessed by a set of observation points (further also referred to as ‘points’) that mimic the service users. Geographical placement of the observation points affects the service assessment as latency is naturally dependent on the distance. A proper distribution of the observation points mitigates the effect of geographical distance. However, this does not solve the problem of incorrect service assessment, as some latency measurements from the observation points are blocked en route. The blocked latency values carry important information and their omission from the statistical processing is detrimental to the result. Blocking of latency measurements is due to the policy of intermediate devices (routers, firewalls) and cannot be regulated. Therefore, it will always be present in Internet communication.

The idea presented in this work is to assess a service by both observed and unobserved latency. The observed latency is the measured true latency. The unobserved latency (also referred to as ‘censored’) is lost due to measurement blocking en route. Therefore, it is only partially known. Survival theory is applied to process blocked latency measurements (Kaplan & Meier, 1958). There is an analogy with healthcare, which is the original field of survival analysis. A studied set of patients with a fatal disease is cured by a new drug or therapy. They are periodically checked by the doctors to observe their lifetime (latency). If the death event occurs, their lifetime is observed. If a patient drops out of the drug study by not visiting the doctors (latency measurement is blocked), the patient’s lifetime is only partially known–it is unobserved. Survival theory allows to assess the lifetime (service latency) of the patients including the unobserved values, as they carry important information for the assessment. The lifetimes of the studied patients can be compared with other patients cured with another drug (or placebo) to observe the effect of the drug. In our application, the drug studied is the Internet service, and the comparison indicates a better service.

There will always be some censoring present in a larger number of patients. This also holds for a larger number of observation points as some latency measurements will be blocked. It has to be noted that the scale of the patient’s lifetime in years and service latency in milliseconds are completely different.

This work finds its use in the general assessment of Internet services. Different services offering the same functionality can be assessed for contracting purposes. Contact points of the same service can be assessed for their automatic selection for users. Furthermore, when services or their contact points are compared, it is important to know the magnitude of the latency difference. This information also helps when other factors are present, such as the cost of service optimization.

**Figure 1:**
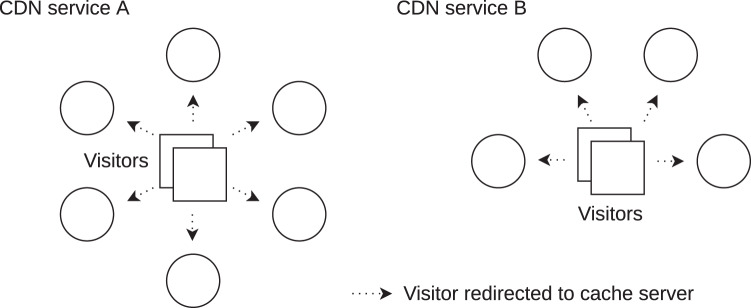
Two CDN services with a different number and geographical placement of their contact points with cached content (cache servers). It is not known which CDN service provides lower latency for e-commerce website visitors from a country.

An example application is CDN service comparison for contracting purposes. CDN services distribute website content closer to visitors for faster page loading (IEEE, 2018). In general, a faster website means more visitors and higher revenues in e-commerce. CDN services have a different number of contact points with cached content. Their geographical distribution in countries varies, and the backbone peering to other networks is also different. An example of two CDN services with different numbers of contact points and their geographical distribution is shown Fig. 1. Using this work, alternative CDN services can be compared for web visitors in a country and sorted by the lowest latency. The best CDN service is contracted on the basis of the magnitude of the latency difference and the price. The method can also be applied to assess free web hosting providers. In this case, the hosting servers of different providers are compared in terms of better latency only.

It has to be noted that the simplified selection of CDN services based on the number of contact points in a country is not correct. It is also not correct to select a service contact point that is geographically closest to the user. Such a selection is not related to the lowest latency. In fact, latency depends on the actual connection between the user and the service contact point. Using the method of survival latency, a set of CDN service contact points can be periodically evaluated and sorted by the lowest latency for users in a country or region. A user is then automatically assigned the true best latency contact point to connect.

The rest of the article is structured as follows: The next section gives an overview of the related work. The ‘Traditional service assessment’ section explains the problem of biased service assessment by latency. The method of survival latency is described in the ‘Survival latency method’ section. Implementation of the method using the RIPE Atlas is described in the ‘Implementation’ section. The ‘Example’ section includes an unbiased service comparison.

## Related work

The importance of latency for Internet services has been studied in a number of works (Briscoe et al., 2016). In addition to technical impacts by Abdou & Oorschot (2017), Gauttam et al. (2022) and user experience by Sharad & Lorch (2009), Demirci et al. (2017), latency has also had a great value in e-commerce (Akamai, 2017).

Service latency is reduced by the presence of contact points that are geographically distributed. With CDN, service users are directed to the nearby contact point, which is a server that offers the service. This redirection is based on the domain name system and global routing. With domain names, the IP address of the service contact point is returned for the service, and the client contacts it on its own. Anycast BGP (Border Gateway Protocol) routing announces the service IP address from more locations, and the client is automatically routed to the service contact point.

CDN providers differ in the number of their contact points, ranging from tens to thousands (Calder et al., 2015). The geographical distribution of the contact points is also different. In Calder et al. (2015), the median distance from the users to the service contact points was: closest 280 km, second closest 700 km, third closest 1,000 km (approx.), and fourth closest 1,300 km. The users were weighted by client Bing query volumes. However, BGP anycast directed only about half of the users to the closest contact point. A total of 75% of the users were directed to a contact point that was within the distance of about 400 km and 90% were directed to a contact point that was within 1,375 km distance. This means that the geographical distance is not only set by the location of the contact points, but also by the Internet routing policy.

Survival theory has already been applied in different fields, including digital communication. Samarakoon et al. (2021) applied survival analysis to evaluate the reliability of wireless connectivity by the probability of channel blocking events. They predicted the maximum transmission duration without failure. The area of interest was ultra-reliable communication for mission-critical 5G applications.

In 3GPP (2021), survival time was introduced as a service parameter. This parameter allows an application to continue without reception of a required message for a specific time. Based on this property, Gebert & Wich (2020) proposed a new scheme of alternating data transmission. When alternating data over two links, a service with a defined survival time related to the message transmission period was proved to be still functional, even though the other link experienced failures.

In Luo, Xu & Bao (2018), the authors proposed a MIMO survival system to model the propagation of radio signals in military scenarios. MIMO systems were found to be suitable for electronic warfare conflicts due to reliable communication. However, MIMO antennas can be physically damaged during service. The antenna survival probability was used to evaluate the loss of channel gain for the outage capacity. The result was that it is more important to protect/guarantee the antennas at the receiver than at the transmitter.

To the best of the author’s knowledge, survival theory has not been applied in Internet service assessment to handle the problem of blocked latency measurements.

## Traditional service assessment

**Figure 2:**
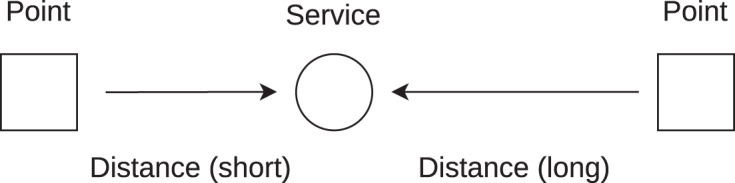
Two observation points measure latency to a service. Latency is affected by different geographical distances.

Traditional service assessment is demonstrated by Fig. 2, which shows latency observation points that mimic service users. This figure and the following ones are plotted from the service point of view, that is, the observation points initiate latency measurements to a service. The service may also initiate latency measurements to the observation points. The direction of latency measurement is not further differentiated as it does not change the method description. The term ‘service’ is used generally as it may refer to many service contact points or a single server through which the Internet service is offered. It may also refer to an end host when its ISP connection service is assessed.

In general, the geographical distance between the observation points and the service is different, as it is shown in Fig. 2. The transmission speed of digital information in optical cables is 2/3 of the speed of light in a vacuum, which is about 200 km/ms (Percacci & Vespignani, 2003). The latency is further increased by the number of routers on the path and by other minor delays related to data transmission (serialization, *etc*.). These delay contributors lower the speed of data transmission to 4/9 of the speed of light in a vacuum, which is 133 km/ms (Katz-Bassett et al., 2006). It has to be noted that with the future development of the Internet, this speed will be increasing towards the theoretical speed of 200 km/ms (Singla et al., 2014).

Taking into account the speed of digital information transmission, a distance of 266 km results in a latency increase by 4 ms, when the round trip time is calculated. The real communication distances between users and services are much larger for non-cached services. They are also larger for cached services, as elaborated in the section ‘Related work’. Therefore, the communication distance has a strong effect on service latency. The effect of distance on service assessment can be mitigated by a set of observation points that mimic the users. These observation points are geographically distributed over the area in question, that is, over the area from which the service users come (further referred to as ‘service area’). In the case of ISP connection service assessment, the observation points mimic the services as the end hosts access them.

**Figure 3:**
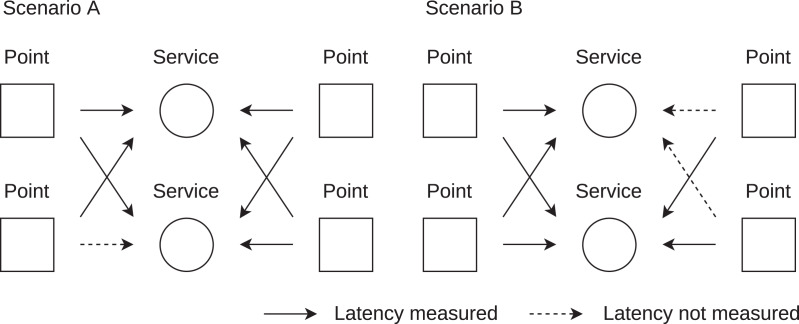
Problem of service assessment by latency. In scenario A, four latency values are observed for the top service, one is missing for the bottom service. In scenario B, two latency values are missing for the top service, four latency values are observed for the bottom service.

With a larger number of observation points, a number of latency measurements will be blocked en route. Figure 3 describes the problem of latency blocking in service assessment. Simply said, the latency of high values may be blocked (not measured) for one service, whilst included in the other service assessment. Moreover, the number of blocked latency measurements can be different for each service. Therefore, statistical processing of the observed latency only leads to incorrect results.

Figure 3 shows the case where the latency of the two services is observed from the same number of observation points. However, this may not be true over time as some points may be temporarily unavailable/abandoned and new observation points may be available. Therefore the number of observation points and blocked latencies can be different for each service assessment.

## Survival latency method

**Figure 4:**
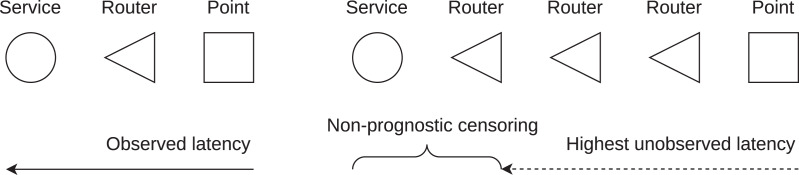
Observed and unobserved (censored) latency measured from observation point to service. Unobserved latency is the maximum latency measured on the path. Non-prognostic censoring is the assumption for survival analysis.

The idea of survival latency is to include the blocked latency measurements in the service latency statistics. Figure 4 shows the basis of observed and unobserved latency on the path from the observation point to the service. A theoretical scenario is shown, the number of routers on the path is not related to the number of observed or unobserved latency values. There is also no relation between the service and the number of routers on the path. The observation point sends an echo message to measure the observed latency. It may happen that the request or response is not communicated. The reason can be the message blocking en route, or other unspecified networking problems. In this case, the unobserved latency is measured to each of the routers on the path. The observed latency is known to be within the interval (*t*_max_, inf), where *t*_max_ is the highest unobserved latency. It has to be noted that the maximum unobserved latency is considered, not the latency measured for the responsive router closest to the service.

Survival theory handles the problem of observed and unobserved data to obtain an unbiased result. The survival function

(1) $$S(l) = P(L > l) = 1 - F(l) = \int_l^\infty f(u)du$$

gives the probability of latency greater than *l*. *L* is a random variable expressing the latency and *F*(*l*) = *P*(*L ≤ l*) is the cumulative distribution function.

The survival function includes both observed and unobserved values. It is obtained using the Kaplan-Meier estimator (Goel, Khanna & Kishore, 2010), which is

(2) $$\hat S(l) = \prod\limits_{{l_i} \le l} \displaystyle{{{n_i} - {d_i}} \over {{n_i},}},$$

where *n*_*i*_ is the number of latency values at risk up to latency *l*_*i*_, *d*_*i*_ is the number of latency values observed at *l*_*i*_, and *l*_*i*_ is the time equal to at least one latency observed.

**Figure 5:**
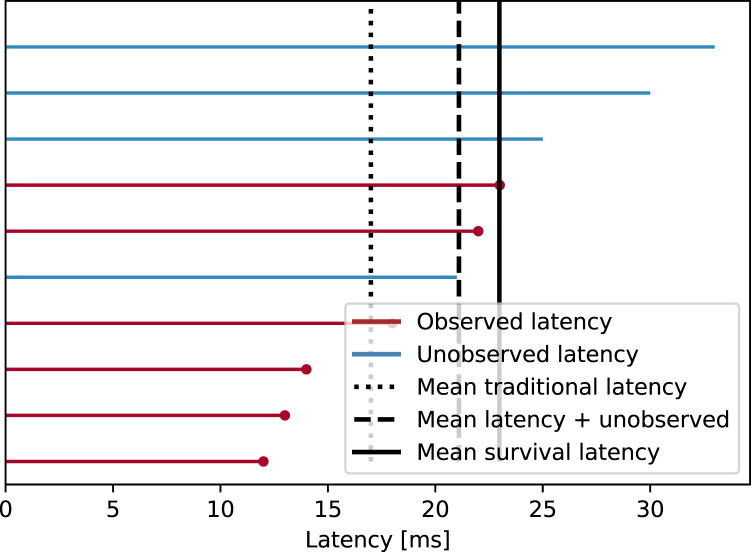
Sample of observed and unobserved latency. Red lines ended by a dot show latency that was measured–observed. Blue lines show latency measurement that was blocked–unobserved. Mean traditional latency shows the value obtained from the observed latencies only. Mean survival latency shows the value obtained from observed and unobserved latencies processed by the survival method.

A service can be assessed by the mean or median survival latency. Median survival latency is the latency with a survival probability of 0.5. However, the median may not be significant for service assessment (unlike in healthcare) as it does not interpret the skewness of the latency values. The mean survival latency is a better metric when the magnitude of the latency difference is important to know. The importance of mean survival for service assessment is demonstrated in Fig. 5, where a limited latency sample is shown for clarity of demonstration. The mean traditional latency, which is taken from the observed values only, is 17 ms. The combination of observed and unprocessed unobserved latency values produces a mean latency of 21 ms. Finally, the mean survival latency is 23 ms, which is defined as the area under the survival curve. The results show that the mean traditional latency underestimates the latency by 6 ms. Therefore, the omission of the unobserved latency results in an inaccurate service assessment. Simple inclusion of the unobserved values still underestimates the result.

In the sample, the mean survival latency was calculated using the whole area under the survival function curve. However, real data have outliers that have a strong effect on the mean survival and thus on the magnitude of the latency difference. Larger latencies (both observed and unobserved) may be a result of temporary outages and their inclusion would be incorrect for service assessment. Such latencies cannot simply be removed from the input data using a latency threshold. The reason is that it is not known which unobserved latency will cross the threshold line and which not. Therefore, the mean survival is found using the restricted mean survival (RMST), which is

(3) $${\rm RMST}(l) = \int_0^l S(u)du.$$

The restricted mean survival uses the limit *l* for the integral to exclude large latency values caused by temporary network problems. The limit selection is further discussed in the section ‘Implementation’.

The steps of the survival latency method are the following: (i) Measure observed latency to service. (ii) Measure unobserved latency to service. (iii) Estimate the survival latency function and calculate the restricted mean. The input prerequisite is a set of latency observation points that are distributed over the service area.

The above steps are processed for each service compared. The time for service assessment is determined by the number of observation points. Therefore, the number of available points can be reduced to limit the measurement time. A service is first approached by an echo message to obtain the observed latency. The observation points, whose latency measurement was blocked, then measure the latency to the routers en route. Parallel processing is possible.

The use of the Kaplan-Meier estimator is conditioned by non-informative censoring (Turkson, Ayiah-Mensah & Nimoh, 2021). Non-informative censoring includes two cases, which are non-prognostic and independent censoring (Leung, Elashoff & Afifi, 1997). The validity of one case is sufficient for non-informative censoring.

- Independent censoring–The observed latency is independent of the unobserved latency. By the nature of latency blocking in the Internet, there is a positive correlation between observed and unobserved latency. The explanation is that latency measurements are typically blocked by routers (firewalls) closer to the destination. Therefore, this assumption is invalid.
- Non-prognostic censoring–An unobserved latency value does not carry prognostic information about the true latency. This assumption is considered valid for Internet communication. We do not have any information about the true latency increase towards the service given the unobserved value, which is the maximum value obtained from the responsive routers on the path.

There are two other assumptions associated with the Kaplan-Meier survival analysis (Goel, Khanna & Kishore, 2010): (i) Survival latency probabilities obtained from observation points that started the measurement at different times are the same. For the service assessment, it is assumed that the measurements start at the same time relative to the service survival prospect; (ii) The events occur at the time measured. This is assumed to be true as we know the exact observed and unobserved latency values.

## Implementation

An implementation is presented to apply the method of survival latency in a real scenario. In particular, the implementation shows that the omission of blocked latency measurements underestimates the service latency. This is detrimental to service comparisons or their optimal setup. In a proper implementation, the latency observation points that mimic the service users must be geographically located in the targeted service area. It may be sufficient to delimit the service area by country borders for national-related services. For international services, a group of target countries or whole continents can be used. The area may also be delimited by other means, such as a country region, a circle with a given radius, or a general polygon. Worldwide coverage is also possible if there is such interest.

**Figure 6:**
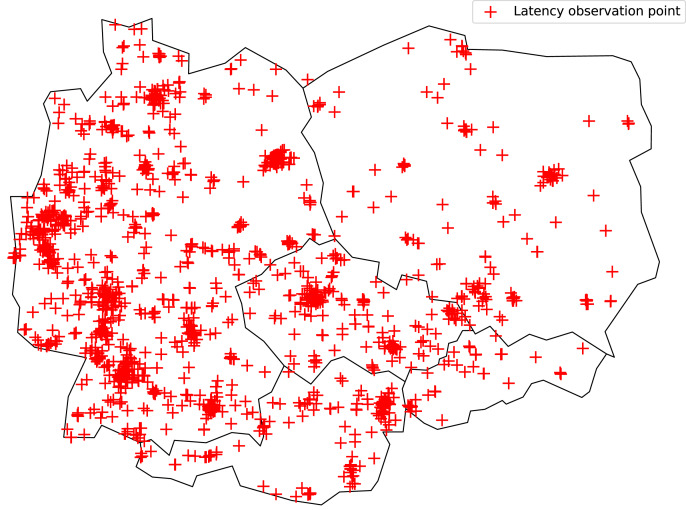
RIPE Atlas observation points in five European countries (Germany, Poland, Czech Rep., Austria, and Slovakia).

In the implementation, the latency observation points that mimic the service users belong to the RIPE Atlas (Bajpai, Eravuchira & Schwnwalder, 2015). The Atlas is a word-wide network of observation points (Candela et al., 2019). As an example, Fig. 6 shows the distribution of observation points in five European countries. Atlas points (probes in Atlas terminology) are dedicated hardware or software running on general devices (RIPE NCC, 2020). Each Atlas point has a public IP address that is used for latency measurement.

ISP connection service of a national operator (Vodafone CZ) is used to demonstrate the service assessment by latency. The latency of the ISP connection is determined by the technology used and by the ISP peering to other networks. Peering is typically implemented in the country of the end host.

**Figure 7:**
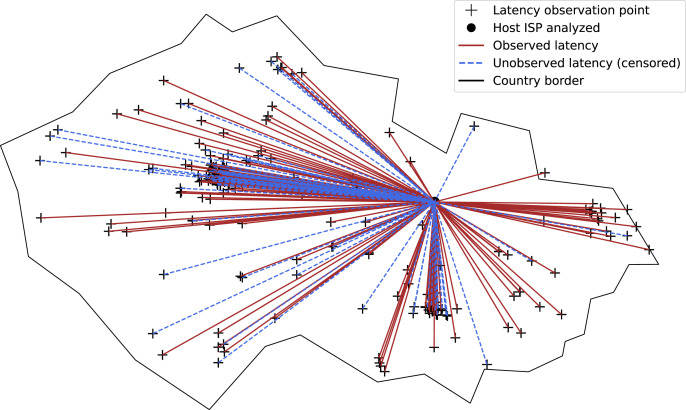
Observed and unobserved latency links of ISP connection service in a country. The location of the end host is artificial for clarity of presentation.

Figure 7 shows the latency observation links used for an ISP connection service. For the sake of clarity, only the link status (observed/unobserved latency) is shown. The location of the end host is artificially set so as not to overlap with the observation points.

**Figure 8:**
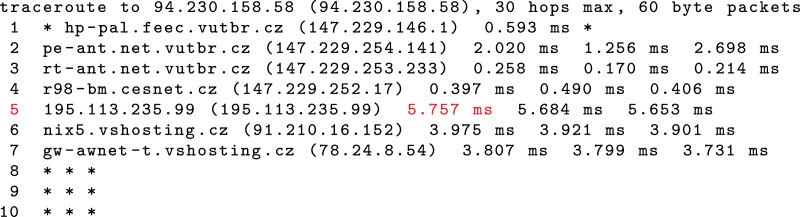
Collection of unobserved latency if the destination is not reached. The case is shown when the unobserved latency is not the value observed for the last responsive router on the path (seventh router), but is the highest latency measured on the path (fifth router).

Latency is measured from the end host to the observation points. First, the *ping* tool is used to collect the observed latency values. Second, the *traceroute* tool is used to collect the unobserved latency values. The unobserved latency is the highest latency measured on the path if the destination was not reached, as shown in Fig. 8.

**Figure 9:**
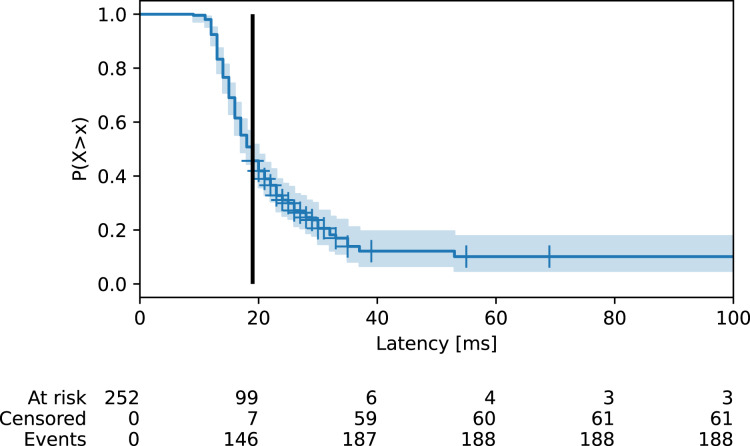
Survival latency function for an ISP connection service. The line shows median survival and crosses show unobserved (censored) latencies. Censoring starts to occur with higher latency values as the measurements are blocked further en route from the end host.

The survival latency function (Eq. (1)) for the ISP connection service is estimated using the Kaplan-Meier estimator (Eq. (2)). The shape of the function is shown in Fig. 9. The figure shows that the survival latency median *M* is obtained graphically as *S*(*M*) = 0.5 from data collected in a country. The tail of the function also demonstrates the need for a maximum latency restriction to exclude erroneous latencies caused by temporal network problems. This is further implemented by restricted mean survival. There were in total 252 latency values, of which 189 were observed and 63 were unobserved. The table below the figure gives an overview of the censoring. The row ‘At risk’ gives the number of remaining latency values in the survival analysis by the end of the interval. The row ‘Censored’ shows the number of unobserved latency values that occurred by the end of the interval. The censored values are also shown as crosses along the curve. It is apparent that the censoring starts to occur with higher latency values. The reason is that the latency measurements are typically blocked further en route, closer to the destination. The row ‘Events’ gives the number of observed latency values that occurred by the end of the interval. The median survival latency for the service is 19 ms and the blue shaded area shows 95% confidence intervals. The function shows a typical tail for latency values in Internet communication. The tail is trimmed for clarity of presentation.

## Example

This section demonstrates the final outcome of the survival latency method, which is unbiased service comparison. The example works with the data from the implementation part that were given for an ISP connection service. These data are extended by additional measurements of a different ISP connection service. The magnitude of the difference in latency between the two ISP connection services is calculated to demonstrate the biased and unbiased comparison.

The latency of each ISP connection service was assessed using the same set of observation points. The limit *l* in Eq. (3) for restricted mean survival was set to 150 ms to exclude the latency values caused by temporary network problems. The limit was also applied in the traditional latency calculation. Table 1 shows the results. The first column shows the mean traditional latency taken from the observed values only. The second column shows the mean latency when the survival method is applied. The mean traditional latency underestimates both ISP connection services, with a larger mismatch of 15 ms for the ISP of host A.

**Table 1:** Mean latency difference between two ISP connection services. Mean latency values areobtained by traditional and survival calculations.

	Traditional latency (ms)	Survival latency (ms)
Host A ISP	18	33
Host B ISP	9	16
Difference	9	17

Both columns show that the ISP connection service of end host B is better. However, when considering the magnitude of the difference, the traditional latency severely underestimates the result. The mean survival latency difference is almost twice the mean traditional latency difference. The true magnitude of the difference is important when other factors are to be considered, such as the service price.

**Figure 10:**
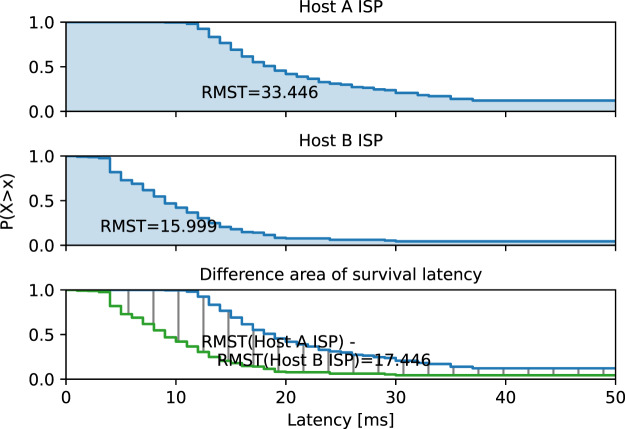
Comparison of two ISP connection service latencies. Latency limit of 150 ms is used to exclude the latency caused by temporary networking problems.

Figure 10 shows graphically how the values in Table 1 in the column ‘Survival latency’ are calculated. The survival latency mean is the area under the survival function curve. Survival latency functions are plotted for each ISP connection service. Both curves are restricted by a value of 150 ms to exclude large latency values caused by temporary network problems. The area difference in the bottom subfigure gives the unbiased comparison value of the two ISPs, which is 17 ms.

## Conclusions

Internet services differ in quality, and latency is one of the key parameters. Naively, service latency is assessed by a single observation point. Such service assessment is biased as the geographical distance between the observation point and the service affects the latency. A set of distributed observation points is used to mitigate the effect of geographical distance. However, some latency measurements from the observation points will be blocked en route, given the policy of intermediate devices, which is detrimental to the service assessment.

The idea of this work was to consider both observed and unobserved (censored) latency for an unbiased service assessment. Survival theory, originally used in healthcare, was used to calculate the restricted mean survival latency. The method can be applied if specific assumptions are met, including non-informative censoring. The validity of the assumptions for Internet communication was discussed.

In addition to service comparison, this work can be used for service optimization or new service setup. A service provider may consider the geographic placement of their servers in an area to achieve a low latency. The magnitude of the difference in latency between different geographic configurations can be used to select the best latency option at a cost. The cost is the number of servers needed and their operation at the selected locations (Lee, Manjunath & Joo, 2022). An example is the optimal server placement for multiplayer online gaming (Benamer, Hadj-Alouane & Boussetta, 2021). In this case, the distribution of the servers should be low-cost from the point of view of the gaming operator. On the other hand, low latency should be achieved for players, as latency is crucial for them.

The method of survival latency was demonstrated by an implementation that evaluates the ISP connection services. This type of service was selected for implementation as it has a straightforward application for general users who are interested in an unbiased assessment of their ISPs.

The raw data and implementation source code are available at Komosny (2022). The published Python notebook with raw data reproduces the results in the article. The notebook with custom data can be used to compare host connection services using the survival latency method and the traditional method with different parameters.

## Short biography

Dan Komosny is a professor at Brno University of Technology, Czech Republic. He received his PhD. degree in Teleinformatics in 2003. His research is focused on IP networks and cybergeography. He lectures courses on operating systems and data networks. He also runs Cisco Networking Academy courses.

## Supplemental Information

10.7717/peerj-cs.1072/supp-1 Supplemental Information 1 Measured latency values of ISP connection service.

## Additional Information and Declarations

### Data Availability

The following information was supplied regarding data availability: Raw data and source code are available at GitHub: https://github.com/komosny/survival-latency. Raw measurements are available as a Supplemental Files.
